# Patterns of Lateral Lymph Node Involvement by Neck Level in cNIb Differentiated Thyroid Carcinoma: A Systematic Review and Meta-Analysis †

**DOI:** 10.3390/diagnostics15202613

**Published:** 2025-10-16

**Authors:** Dana M. Hartl, Karthik N. Rao, Andrés Coca Pelaz, Alessandra Rinaldo, Mark E. Zafereo, Greg W. Randolph, Iain J. Nixon, Marc Hamoir, K. Thomas Robbins, Luiz P. Kowalski, Pia Pace Asciak, Badr Soudi, Juan P. Rodrigo, Alfio Ferlito

**Affiliations:** 1 Division of Surgery and Anesthesiology, Head and Neck Oncology Service, Thyroid Surgery Unit, Gustave Roussy Cancer Campus Grand Paris, 94800 Villejuif, France; soudi.badr@uiass.ma; 2 Department of Head and Neck Oncology, Sri Shankara Cancer Foundation, Bangalore 560004, India; karthik.nag.rao@gmail.com; 3 Department of Otolaryngology, Hospital Universitario Central de Asturias, ISPA, IUOPA, CIBERONC, University of Oviedo, 33003 Oviedo, Spainjprodrigo@uniovi.es (J.P.R.); 4 ENT Unit, Policlinico Città di Udine, 33100 Udine, Italy; dottalerinaldo@gmail.com; 5 Head and Neck Endocrine Surgery, MD Anderson Cancer Center, The University of Texas, Houston, TX 77030, USA; mzafereo@mdanderson.org; 6 Division of Thyroid and Parathyroid Endocrine Surgery, Department of Otolaryngology, Massachusetts Eye and Ear Infirmary, Boston, MA 02114, USA; gregory_randolph@meei.harvard.edu; 7 Department of Otolaryngology Head and Neck Surgery, University of Edinburgh, Edinburgh EH8 9YL, UK; iain.nixon@edinburghent.com; 8 Department of Otorhinolaryngology, Head and Neck Surgery, King Albert II Cancer Institute, Cliniques Universitaires Saint-Luc, Institute de Recherche Expérimentale, 1200 Brussels, Belgium; marc.hamoir@saintluc.uclouvain.be; 9 Department of Otolaryngology-Head and Neck Surgery, Southern Illinois University School of Medicine, Springfield, IL 62702, USA; kthomasrobbins@gmail.com; 10 Department of Otorhinolaryngology-Head and Neck Surgery, A.C. Camargo Cancer Center, São Paulo 01509-010, Brazil; lp_kowalski@uol.com.br; 11 Department of Head and Neck Surgery, University of São Paulo Medical School, São Paulo 05403-000, Brazil; 12 Department of Otolaryngology—Head and Neck Surgery, St. Joseph’s Health Centre, University of Toronto, Toronto, ON M5S 1A1, Canada; piapaceasciak@gmail.com; 13 Coordinator of the International Head and Neck Scientific Group, 35100 Padua, Italy; profalfioferlito@gmail.com

**Keywords:** papillary thyroid carcinoma, lymph node metastases, lateral neck dissection, cervical lymph nodes, differentiated thyroid cancer

## Abstract

**Background/Objectives:** The optimal extent of lateral lymph node dissection in cN1b differentiated thyroid cancer remains controversial. This systematic review aimed to assess the frequency of lymph node involvement across neck levels I to V. **Materials and Methods:** A systematic review was conducted following PRISMA guidelines. PubMed was searched for studies on lateral neck dissection in differentiated thyroid cancer. Included studies reported level-specified metastatic rates. Data on patient numbers and metastatic events were extracted. A random-effects meta-analysis with Freeman–Tukey double arcsine transformation was performed for each neck level to calculate pooled prevalence proportions and 95% confidence intervals. Heterogeneity was assessed using the I^2^ statistic. **Results:** Meta-analysis of 57 studies revealed that level III (68%, 95% CI: 63–73) and level IV (66%, 95% CI: 61–70) had the highest metastatic prevalence, followed by level IIA (46%, 95% CI: 37–56). Level V demonstrated an overall prevalence of 22% (95% CI: 18–26), with sublevel VB (19%, 95% CI: 11–28) significantly higher than VA (4%, 95% CI: 1–9). Level I (6%, 95% CI: 2–11) and sublevel IIB (14%, 95% CI: 9–20) showed the lowest risk. Significant heterogeneity (I^2^ 71–94%) was observed across all levels. **Conclusions:** Our findings support sparing level I, and sublevels IIB and VA during lateral neck dissection. Current guidelines recommend systematic dissection of IIA, III, IV, and VB, although VB involvement was found to be only 19% in our study. Future personalization of the extent of neck dissection, based on individual risk factors, may be key to optimizing oncologic and functional outcomes.

## 1. Introduction

Differentiated thyroid carcinoma (DTC) is the most common endocrine malignancy, with a rising incidence worldwide. Although DTC has an excellent prognosis, lymph node metastases are frequent, occurring in 30–80% of cases [1]. Patients with lateral neck metastases (cN1b) present a particular challenge, as these metastases, while having a limited impact on overall survival, significantly increase the risk of regional recurrence and can therefore negatively affect patients’ quality of life [2,3].

The management of lymph node metastases involves lateral neck dissection; however, the optimal extent of this dissection remains a topic of debate, particularly concerning sublevels IIB and VA. Sublevels IIA, IIB, VA, and VB correspond to anatomical subdivisions of levels II and V, which are clinically relevant due to their variable risk of metastases and distinct surgical morbidity. This systematic review aims to examine the frequency of metastasis across different neck levels and sublevels to aid in rationalizing the extent of therapeutic neck dissection.

## 2. Materials and Methods

This study was reported in alignment with the PRISMA (Preferred Reporting Items for Systematic Reviews and Meta-Analyses) guidelines [4]. The PRISMA checklist can be consulted in the Supplementary Material. A systematic literature search was conducted of the PubMed database from January 1983 through February 2025. The search strategy included the following terms: “neck dissection”, “lymph node metastases”, “lymph node involvement”, “thyroid cancer”, “papillary thyroid cancer”. Boolean operators (AND, OR) were used to refine the search results.

The included studies focused on differentiated thyroid cancer with pathologically proven lateral cervical lymph node metastases in adult patients and were published in English. Levels and sublevels (I, II [IIA/IIB], III, IV, and V [VA/VB]) were defined according to the American Academy of Otolaryngology–Head and Neck Surgery classification [5].

Exclusion criteria encompassed case reports, studies focused on non-papillary thyroid cancer or non-differentiated thyroid cancer, those that did not specify the frequency of lateral thyroid metastases or provide level-specific data, and studies addressing only central neck dissection or recurrent thyroid cancer. Studies not using the standardized nomenclature for lateral neck node levels as defined by the American Academy of Otolaryngology–Head and Neck Surgery and adopted by the American Thyroid Association [1,6] were also excluded.

**Scheme 1:**
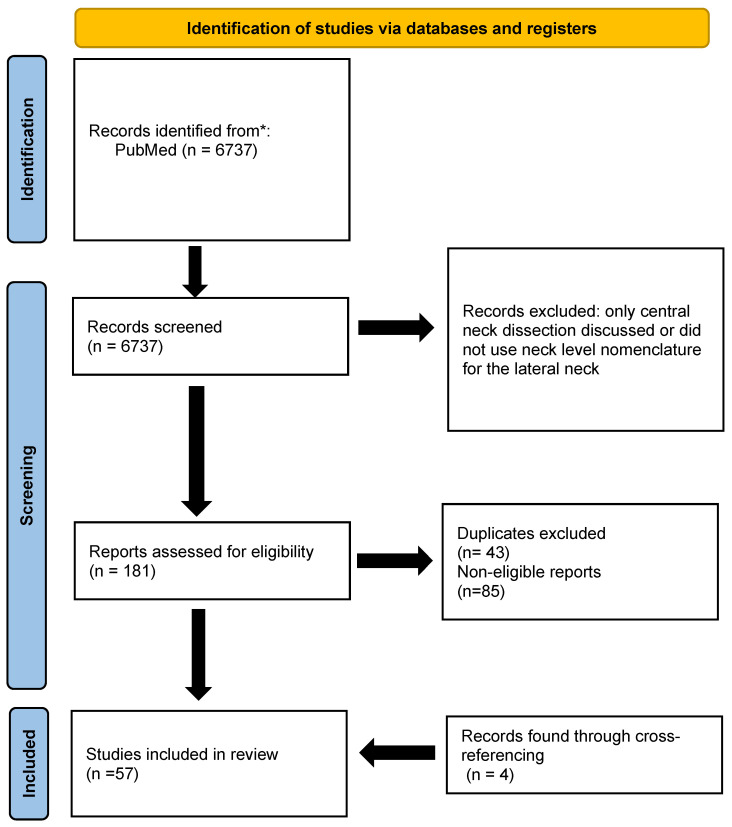
PRISMA 2020 flow diagram for new systematic reviews which included searches of databases and registers only. * Automation tools were not used. Source: Page, M.J. et al. BMJ 2021, 372, n71. https://doi.org/10.1136/bmj.n71 [4]. This work is licensed under CC BY 4.0. To view a copy of this license, visit https://creativecommons.org/licenses/by/4.0/. accessed on 20 February 2025.

Two investigators independently screened the retrieved articles based on the type of article, title, and abstract (Scheme 1). Eligible articles underwent full-text review and reference screening using a snowball search method. Any disagreements regarding inclusion were resolved by consensus or consultation with a senior author(s).

Data extraction was performed manually, focusing on the percentage of pathologically proven metastatic involvement at each cervical level. For each lateral neck level (I–V) and sublevel (IIA, IIB, VA, VB), we recorded the number of patients, number of events (metastatic nodes), and corresponding percentages as reported in the final pathology reports. Database searching and data extraction were manually performed. Reported risk factors for lymph node metastases in the different levels and sublevels were also collected.

A meta-analysis was conducted for each neck level and sublevel using a random-effects model with the inverse variance method and Freeman–Tukey double arcsine transformation to stabilize variances [7]. The summarized proportions with 95% confidence intervals were calculated. Heterogeneity was assessed using the Cochran Q-test and I^2^ statistic, with I^2^ values ≥ 50% indicating substantial heterogeneity [8]. Between-study variance was estimated using tau^2^ and tau.

Publication bias was evaluated using funnel plots and Egger’s regression test for asymmetry. Influence diagnostics were conducted using studentized residuals and Cook’s distance [9]. All statistical analyses were performed using R Statistical Software (v4.3.1) with the meta package [10]. The number of studies, total subjects, and summary estimates for each level are reported in the accompanying (Supplementary Material).

**Figure 1:**
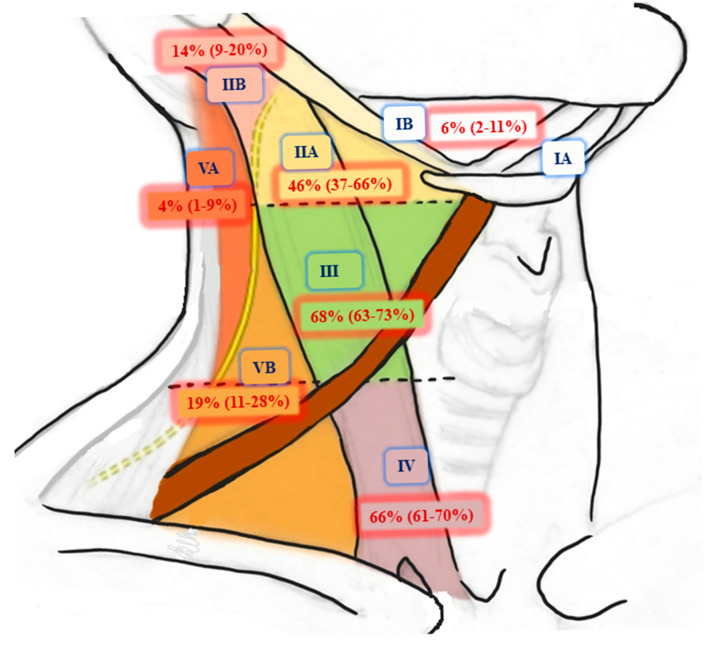
Heatmap of lateral neck nodal metastasis.

A heat map illustrating the distribution of neck nodal metastases was generated using the meta-analyzed data (Figure 1).

## 3. Results

The systematic database search of PubMed yielded 6737 articles. After the removal of 43 duplicates, 6694 records were screened based on title and abstract. During this phase, 6513 records were excluded as they focused only on central neck dissection or did not use the standard AAO-HNS nomenclature for lateral neck levels. The full texts of the remaining 181 articles were assessed for eligibility. A further 124 articles were excluded for reasons including insufficient level-specific data, pooled analysis of neck levels, or not meeting inclusion criteria. Four additional studies were identified through cross-referencing. Ultimately, 57 studies were included in the final quantitative meta-analysis (Table 1).

The qualitative synthesis included data from these 57 studies, reporting on pathological outcomes from a cumulative total of over 17,000 lateral neck levels dissected across levels I through V. All but two of the eligible studies were retrospective cohort studies [11,12].

**Table 1:** Summary of the data in this systematic review.

Neck Level	Author (Reference)	Year	Number of Patients	Events	Percentage (%)
**Level I**	Sivanandan et al. [13]	2001	70	2	4.00%
	Pingpank et al. [14]	2002	51	6	12.00%
	Kupferman et al. [15]	2004	444	62	14.00%
	Amarasinghe et al. [16]	2007	79	7	8.50%
	Roh et al. [17]	2008	52	2	3.70%
	Spriano et al. [18]	2009	77	3	4.00%
	Ahmadi et al. [19]	2011	25	0	0.00%
	Nam et al. [11]	2013	176	0	0.00%
	Eweida et al. [12]	2017	30	4	14.00%
**Level II**	Sivanandan et al. [13]	2001	70	35	50.00%
	Kupferman et al. [15]	2004	44	23	52.00%
	Lee et al. [20]	2007	46	28	60.00%
	Amarasinghe et al. [16]	2007	79	12	15.30%
	Ahn et al. [21]	2008	37	24	65.00%
	Lee et al. [22]	2008	167	93	55.50%
	Koo et al. [23]	2009	76	40	52.60%
	Farrag et al. [24]	2009	53	35	66.00%
	Spriano et al. [18]	2009	77	29	38.00%
	Keum et al. [25]	2012	72	41	56.50%
	Merdad et al. [26]	2012	185	91	49.30%
	Park et al. [27]	2012	147	74	50.30%
	Wu et al. [28]	2012	78	45	58.00%
	Zhang et al. [29]	2013	330	215	65.30%
	Nam et al. [11]	2013	175	70	40.00%
	Shim et al. [30]	2013	143	72	50.30%
	Kang et al. [31]	2014	209	112	53.60%
	O’Neill et al. [32]	2014	121	68	56.00%
	Javid et al. [33]	2016	191	131	68.80%
	Yang et al. [34]	2016	220	101	45.90%
	Kim et al. [35]	2017	658	367	55.80%
	Lombardi et al. [36]	2018	405	194	48.00%
	Gong et al. [37]	2018	246	113	45.90%
	Liu et al. [38]	2019	966	408	42.20%
	Li et al. [39]	2020	252	108	42.80%
	Song et al. [40]	2022	134	60	44.80%
	Song et al. [41]	2022	156	67	42.70%
**Sublevel IIA**	Roh et al. [42]	2008	52	38	72.20%
	Koo et al. [23]	2009	76	40	52.60%
	Vayisoglu et al. [43]	2010	33	9	27.20%
	King et al. [44]	2011	32	16	49.00%
	Ahmadi et al. [19]	2011	25	13	50.00%
	Lim et al. [45]	2012	90	42	47.00%
	Kim et al. [46]	2012	18	8	46.70%
	Kim et al. [47]	2016	327	175	53.50%
	Prstačić et al. [48]	2020	53	27	50.80%
	Liu et al. [49]	2020	828	188	22.70%
	Liu et al. [50]	2022	203	81	39.90%
**Sublevel IIB**	Pingpank et al. [14]	2002	51	11	21.00%
	Lee et al. [20]	2007	55	12	22.00%
	Lee et al. [22]	2008	167	11	6.80%
	Roh et al. [17]	2008	52	9	16.70%
	Yanir and Dowek et al. [51]	2008	27	2	7.00%
	Koo et al. [23]	2009	76	9	11.80%
	Farrag et al. [24]	2009	53	5	8.50%
	Vayisoglu et al. [43]	2010	33	1	4.50%
	King et al. [44]	2011	32	20	61.50%
	Kim et al. [35]	2012	18	4	20.00%
	Lim et al. [45]	2012	90	9	10.00%
	Yu et al. [52]	2012	47	17	36.10%
	Kim et al. [47]	2016	327	34	10.40%
	Lombardi et al. [36]	2018	405	24	6.00%
	Prstačić et al. [48]	2020	53	4	8.20%
	Liu et al. [49]	2020	954	137	14.40%
	Hosokawa et al. [53]	2021	41	3	7.30%
	Song et al. [41]	2022	156	15	9.50%
**Level III**	Sivanandan et al. [13]	2001	70	44	63.00%
	Kupferman et al. [15]	2004	44	25	57.00%
	Lee et al. [20]	2007	46	38	82.00%
	Amarasinghe et al. [16]	2007	79	11	13.60%
	Roh et al. [17]	2008	52	38	72.20%
	Yanir and Dowek et al. [51]	2008	27	18	68.00%
	Ahn et al. [21]	2008	37	30	80.00%
	Koo et al. [23]	2009	76	55	72.40%
	Farrag et al. [24]	2009	53	35	66.00%
	Spriano et al. [18]	2009	77	35	45.00%
	Vayisoglu et al. [43]	2010	33	18	54.50%
	King et al. [44]	2011	18	13	71.70%
	Ahmadi et al. [19]	2011	25	14	57.00%
	Merdad et al. [26]	2012	185	142	76.60%
	Keum et al. [25]	2012	72	50	69.40%
	Lim et al. [45]	2012	90	68	76.00%
	Park et al. [27]	2012	147	129	87.80%
	Wu et al. [28]	2012	78	51	65.00%
	Kim et al. [35]	2012	490	262	53.50%
	Nam et al. [11]	2013	176	81	46.00%
	Zhang et al. [29]	2013	330	259	78.40%
	Shim et al. [30]	2013	143	114	79.70%
	O’Neill et al. [32]	2014	121	96	79.00%
	Javid et al. [33]	2016	191	125	65.70%
	Yang et al. [34]	2016	220	138	62.70%
	Kim et al. [47]	2016	327	258	78.90%
	Eweida et al. [12]	2017	30	17	58.30%
	Kim et al. [35]	2017	658	482	73.30%
	Lombardi et al. [36]	2018	405	296	73.00%
	Gong et al. [37]	2018	246	154	62.60%
	Liu et al. [38]	2019	966	645	66.80%
	Li et al. [39]	2020	252	179	71.20%
	Prstačić et al. [48]	2020	53	38	72.10%
	Liu et al. [50]	2022	203	171	84.20%
	Song et al. [41]	2022	156	132	84.30%
**Level IV**	Sivanandan et al. [13]	2001	70	39	55.00%
	Kupferman et al. [15]	2004	44	18	41.00%
	Lee et al. [20]	2007	46	35	75.00%
	Amarasinghe et al. [16]	2007	79	24	30.50%
	Roh et al. [17]	2008	52	39	75.90%
	Lee et al. [22]	2008	167	125	74.90%
	Yanir and Dowek et al. [51]	2008	27	15	57.00%
	Ahn et al. [21]	2008	37	28	76.00%
	Koo et al. [23]	2009	76	55	72.40%
	Farrag et al. [24]	2009	53	27	50.00%
	Spriano et al. [24]	2009	77	40	52.00%
	Vayisoglu et al. [43]	2010	33	27	81.80%
	King et al. [44]	2011	18	12	66.60%
	Ahmadi et al. [19]	2011	25	12	46.00%
	Yu et al. [52]	2012	47	36	76.60%
	Keum et al. [25]	2012	72	54	75.00%
	Merdad et al. [26]	2012	185	114	61.60%
	Lim et al. [45]	2012	90	61	68.00%
	Kim et al. [46]	2012	18	13	73.30%
	Park et al. [27]	2012	147	80	54.40%
	Wu et al. [28]	2012	78	57	73.00%
	Nam et al. [11]	2013	176	74	42.00%
	Zhang et al. [29]	2013	330	233	70.60%
	Shim et al. [30]	2013	143	107	74.80%
	O’Neill et al. [32]	2014	121	90	74.00%
	Javid et al. [33]	2016	191	99	52.00%
	Yang et al. [34]	2016	220	122	55.50%
	Kim et al. [47]	2016	327	247	75.50%
	Kim et al. [35]	2017	658	506	76.90%
	Lombardi et al. [36]	2018	405	271	67.00%
	Gong et al. [37]	2018	246	138	56.10%
	Liu et al. [38]	2019	966	650	67.30%
	Prstačić et al. [48]	2020	53	36	67.20%
	Li et al. [39]	2020	252	214	85.10%
	Liu et al. [50]	2022	203	122	60.00%
	Song et al. [41]	2022	156	130	83.10%
**Level V**	Sako et al. [54]	1985	56	31	55.00%
	Sivanandan et al. [13]	2001	70	21	30.00%
	Pingpank et al. [14]	2002	51	14	28.00%
	Kupferman et al. [15]	2004	44	9	20.00%
	Caron et al. [55]	2006	31	20	65.00%
	Lee et al. [20]	2007	46	9	20.00%
	González et al. [56]	2007	60	1	2.00%
	Amarasinghe et al. [16]	2007	79	17	22.00%
	Lee et al. [22]	2008	167	28	16.80%
	Kupferman et al. [15]	2008	70	37	53.00%
	Yanir and Dowek et al. [51]	2008	27	5	20.00%
	Ahn et al. [21]	2008	37	6	15.00%
	Khafif et al. [57]	2008	37	5	13.50%
	Farrag et al. [24]	2009	53	21	40.00%
	Koo et al. [23]	2009	76	12	15.80%
	Iqbal et al. [58]	2009	38	4	11.00%
	Spriano et al. [18]	2009	77	6	8.00%
	Vayisoglu et al. [43]	2010	33	6	18.00%
	Yüce et al. [59]	2010	61	21	34.00%
	Vergez et al. [60]	2010	90	9	10.00%
	Ahmadi et al. [19]	2011	25	5	21.00%
	Keum et al. [25]	2012	72	15	20.80%
	Park et al. [27]	2012	147	17	11.60%
	Lim et al. [45]	2012	90	15	17.00%
	Wu et al. [28]	2012	78	25	32.00%
	Zhang et al. [29]	2013	330	95	28.80%
	Shim et al. [30]	2013	143	26	18.20%
	Nam et al. [11]	2013	176	18	10.00%
	Kang et al. [31]	2014	209	53	25.40%
	O’Neill et al. [32]	2014	121	46	38.00%
	Javid et al. [33]	2016	191	32	16.90%
	Yang et al. [34]	2016	220	27	12.30%
	Kim et al. [47]	2016	327	45	13.80%
	Kim et al. [35]	2017	658	93	14.10%
	Lombardi et al. [36]	2018	405	142	35.00%
	Gong et al. [37]	2018	246	29	11.80%
	Wang et al. [61]	2019	1037	221	21.30%
	Liu et al. [38]	2019	966	206	21.30%
	Prstačić et al. [48]	2020	53	16	31.10%
	Liu et al. [62]	2020	208	37	17.80%
	Li et al. [39]	2020	252	45	17.80%
	Li et al. [63]	2021	132	14	10.60%
	Kang et al. [31]	2022	110	17	15.00%
**Sublevel VA**	Roh et al. [17]	2008	52	0	0.00%
	Farrag et al. [24]	2009	53	0	0.00%
	Lim et al. [45]	2010	70	8	11.40%
	King et al. [44]	2011	39	3	7.60%
	Kim et al. [46]	2012	18	1	6.70%
	Song et al. [64]	2022	46	0	0.00%
	Song et al. [40]	2022	134	8	5.80%
**Sublevel VB**	Sivanandan et al. [13]	2001	70	23	28.80%
	Roh et al. [17]	2008	52	2	3.70%
	Yanir and Dowek et al. [51]	2008	27	2	7.10%
	Farrag et al. [24]	2009	53	21	40.00%
	Lim et al. [45]	2010	70	4	5.70%
	King et al. [44]	2011	39	12	30.70%
	Merdad et al. [26]	2012	185	54	29.20%
	Kim et al. [46]	2012	18	1	6.70%
	Zhang et al. [29]	2013	330	95	28.80%
	Lombardi et al. [36]	2018	405	126	31.00%
	Song et al. [64]	2022	46	14	30.40%
	Liu et al. [50]	2022	203	18	8.90%
	Song et al. [41]	2022	156	9	5.60%

### Prevalence of Metastases by Neck Level

**Figure 2:**
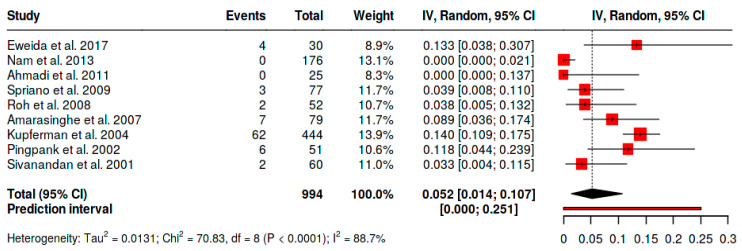
Forest plot for level I nodal metastasis [11,12,13,14,15,16,17,18,19].

**Figure 3:**
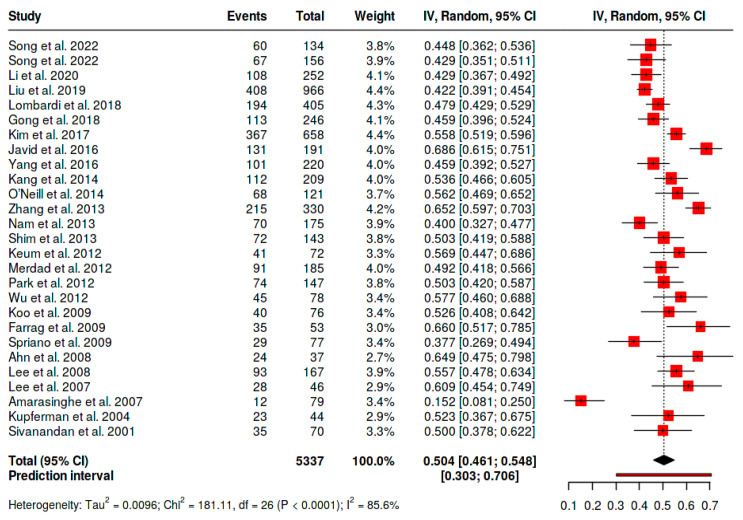
Forest plot for level II nodal metastasis [11,13,15,16,18,20,21,22,23,24,25,26,27,28,29,30,31,32,33,34,35,36,37,38,39,40,41].

**Figure 4:**
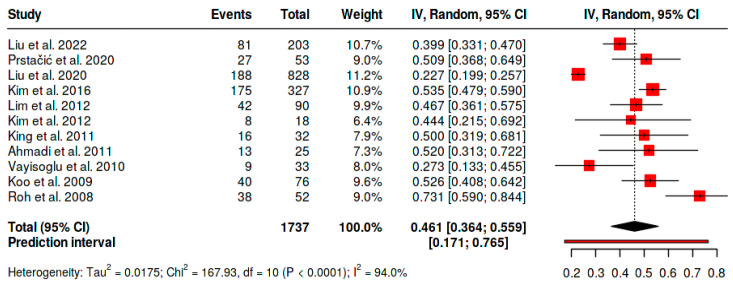
Forest plot for level IIA nodal metastasis [17,19,23,35,43,44,45,47,48,49,50].

**Figure 5:**
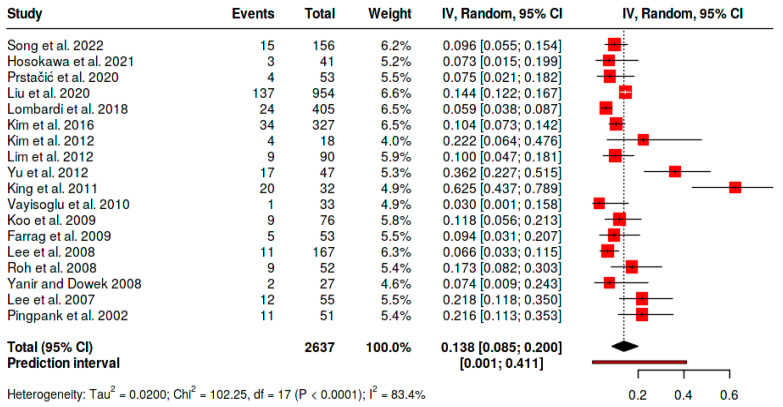
Forest plot for level IIB nodal metastasis [14,17,20,22,23,24,35,36,41,43,44,45,47,48,49,51,52,53].

**Figure 6:**
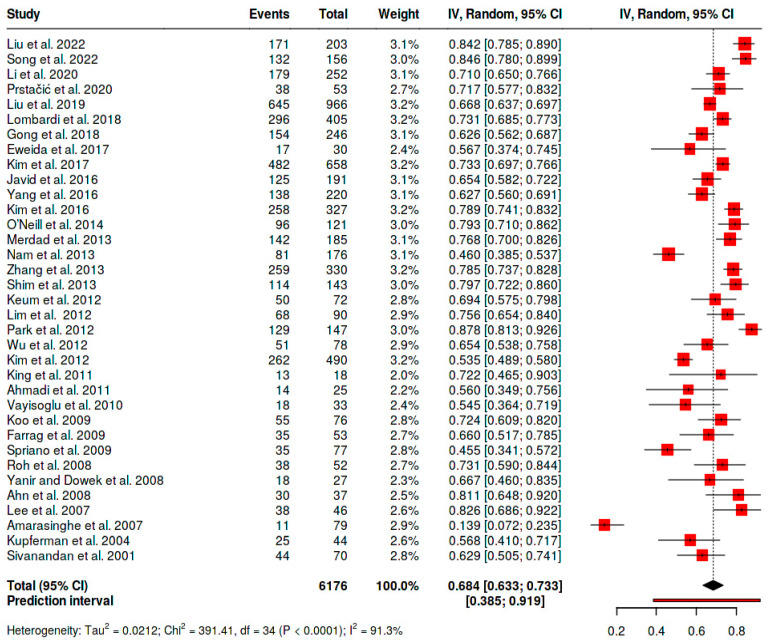
Forest plot for level III nodal metastasis [11,12,13,15,16,17,18,19,20,21,23,24,25,26,27,28,29,30,32,33,34,35,36,37,38,39,40,43,44,45,46,47,48,50,51].

**Figure 7:**
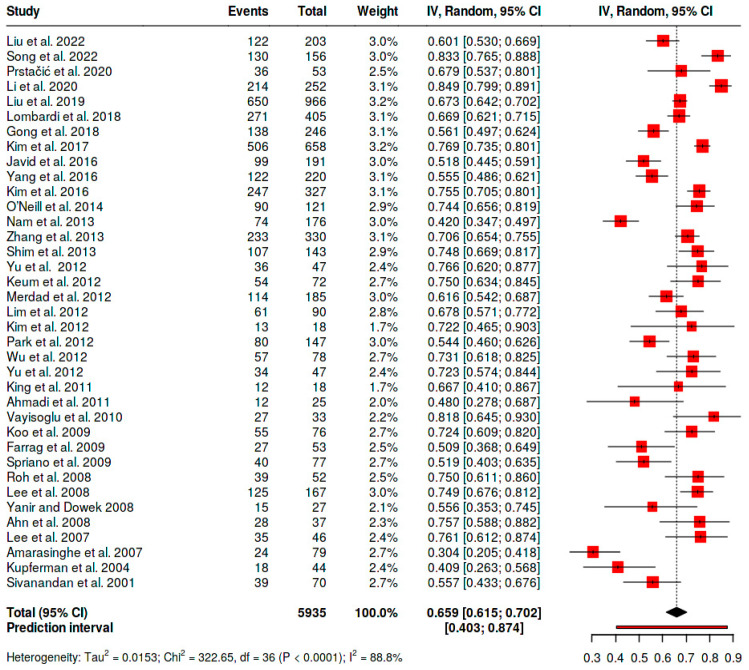
Forest plot for level IV nodal metastasis [11,13,15,16,18,19,20,21,22,23,24,25,26,27,28,29,30,32,33,34,35,36,37,38,39,40,42,43,44,45,46,47,48,50,51,52].

**Figure 8:**
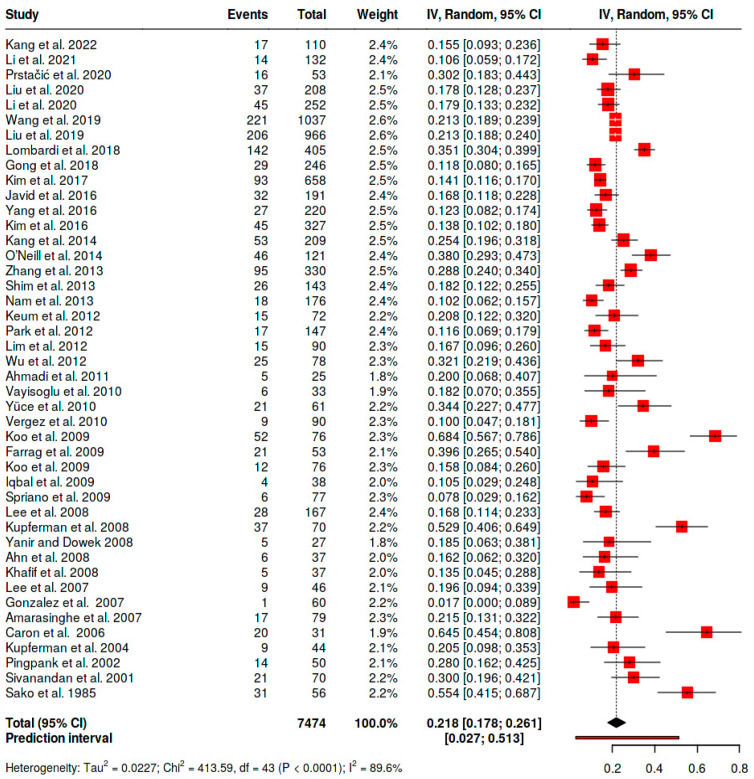
Forest plot for level V nodal metastasis [11,14,15,16,18,19,20,21,22,23,25,27,28,29,30,31,32,33,34,35,36,37,38,39,43,45,47,48,49,51,54,55,56,57,58,59,60,61,63].

**Figure 9:**
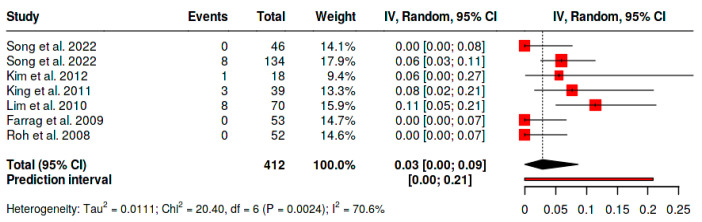
Forest plot for level VA nodal metastasis [17,24,40,41,45,46].

**Figure 10:**
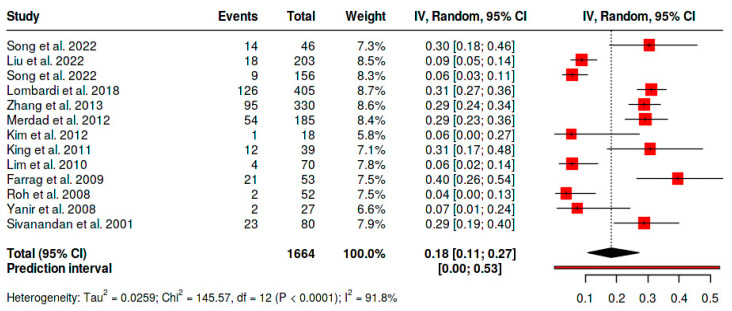
Forest plot for level VB nodal metastasis [13,17,24,26,29,36,40,41,44,45,46,50,51].

A meta-analysis of the extracted data was performed for each neck level and sublevel. Figure 1 illustrates the proportions of involvement of each neck level and sublevel (Figure 1). The summarized proportion of metastatic involvement for each level, calculated using a random-effects model, is reported below. All analyses showed significant heterogeneity (*p* < 0.01), indicating substantial variation in effect sizes across studies (Figure 2, Figure 3, Figure 4, Figure 5, Figure 6, Figure 7, Figure 8, Figure 9 and Figure 10).

Meta-analysis revealed a summarized proportion of metastatic involvement of 6% (95% CI: 0.02–0.11) for level I, based on data from 9 studies encompassing 994 subjects, with significant heterogeneity observed (I^2^ = 89%) (Figure 2). For level II, analysis of 27 studies (*n* = 5337 subjects) showed a metastatic prevalence of 50% (95% CI: 0.46–0.55; I^2^ = 86%) (Figure 3). Subset analysis of this level indicated a higher involvement in sublevel IIA, at 46% (95% CI: 0.37–0.56; 11 studies, *n* = 1737; I^2^ = 94%) (Figure 4), compared to sublevel IIB, which had a prevalence of 14% (95% CI: 0.09–0.20; 18 studies, *n* = 2637; I^2^ = 83%) (Figure 5).

The most frequently involved levels were level III and level IV. The summarized proportion for level III was 68% (95% CI: 0.63–0.73), derived from 35 studies with 6176 subjects (I^2^ = 91%) (Figure 6). Similarly, level IV had a prevalence of 66% (95% CI: 0.61–0.70), based on 37 studies and 5935 subjects (I^2^ = 89%) (Figure 7).

Analysis of level V, which included 44 studies (*n* = 7474 subjects), found an overall metastatic involvement of 22% (95% CI: 0.18–0.26; I^2^ = 90%) (Figure 8). Further subset analysis demonstrated a stark difference between its subdivisions; sublevel VB had a notably higher prevalence of 19% (95% CI: 0.11–0.28; 13 studies, *n* = 1664; I^2^ = 92%) (Figure 10), while involvement in sublevel VA was the lowest of all sublevels analyzed, at just 4% (95% CI: 0.01–0.09; 7 studies, *n* = 412; I^2^ = 71%) (Figure 9).

## 4. Heterogeneity and Publication Bias

Significant heterogeneity was detected in all analyses (*p* < 0.01), with I^2^ values ranging from 71% to 94%, indicating that the vast majority of variability across studies was due to clinical or methodological heterogeneity rather than chance. The between-study variance (tau^2^) was quantified for each level. Funnel plots and Egger’s regression tests did not indicate significant publication bias for any neck level (*p*-values for Egger’s test ranged from 0.258 to 0.681) (Supplementary Materials).

## 5. Discussion

When considering surgical management of the lateral neck in differentiated thyroid cancer, the general consensus is to perform neck dissection (therapeutic neck dissection) when there is preoperatively detected macroscopic disease, also termed clinically apparent disease. However, the extent of neck dissection varies among publications and remains a subject of debate.

Historic approaches included “berry picking” which led to high recurrence rates and has been replaced with a compartment oriented approach. There is a lack of consensus however on the levels that should be dissected. Clearly those involved on pre-operative imaging should be addressed, but the need for prophylactic neck dissection of uninvolved neck levels is not clear. In the lateral neck, most experts and recommendations agree that dissection of the major levels from IIA-VB should be dissected if the lateral neck is involved with disease, regardless of the size of the metastatic nodes or the extent of involvement of the lateral neck.

In squamous cell carcinoma, a threshold of 20% occult involvement is considered a trigger for elective neck dissection. Such a threshold has never been formally agreed upon in differentiated thyroid cancer and it is possible that this figure could be higher given the more indolent nature of the disease and the lack of definite clinical progression of disease in many occult small volume lymph nodes. The rate of pathologic positive but microscopically involved nodes in thyroid cancer remains high, however.

Our review suggests that lateral neck levels IIA-IV harbor metastatic disease at levels well above this 20% threshold. However, levels IIB, VA and VB were involved in 14%, 4% and 19% of cases, respectively. These figures suggest that while level VB (caudal to the accessory nerve) may be considered in a therapeutic neck dissection, 19% being close to the 20% threshold, those levels cranial to the accessory nerve (IIB and VA) are less commonly involved and may be spared if clinically uninvolved. Thus, the validity of the “20% rule” in thyroid cancer remains to be justified and the clinical significance of a 19% involvement rate of level VB may raise concern about overtreatment of this neck level.

Certain risk factors have been shown to be associated with a higher risk of involvement of specific neck levels. Table 2 shows a number of reported risk factors for lymph node metastases in the various neck levels (Table 2). These and potentially other factors could eventually be taken into account when determining the extent of neck dissection in a given patient.

Several current guidelines recommend systematic dissection of sublevel IIA, levels III-IV, and sublevel VB in cN1b papillary thyroid carcinoma, irrespective of nodal location, size, patient age, or tumor subtype. This implies including clinically unaffected adjacent lymph node levels in the therapeutic neck dissection. The American Thyroid Association (ATA) consensus statement is in favor of a comprehensive neck dissection of at least sublevel IIA, levels III, IV, and sublevel VB to optimize disease control, with removal of sublevels IIB and VA only if suspicious nodes are detected in those levels, to minimize complications. (1) The newest ATA management guidelines for adult patients with differentiated thyroid cancer have retained this recommendation to “typically” include levels and sublevels IIA, III, IV and VB, in patients with cN1b disease. This is a “strong” recommendation but based on “moderate certainty evidence”. (2) This recommendation is unchanged from the previous guidelines. (3) Likewise, the British Association of Endocrine and Thyroid Surgeons (BAETS) recommends a selective lateral neck dissection of sublevels IIA–VB), without systematic dissection of levels I, IIB or VA [72].

However, other guidelines, such as that of the National Comprehensive Cancer Network (NCCN) are in favor of a therapeutic neck dissection only of involved neck compartments [73]. The European Society of Medical Oncology (EMSO) is less specific in terms of the extent of neck dissection, recommending a therapeutic lateral neck dissection for patients with biopsy-proven metastatic lateral cervical lymphadenopathy (ESMO) [74]. This leaves the possibility for a more tailored approach, personalizing the neck dissection in terms of extent and risk of morbidity.

The Japanese Association of Endocrine Surgeons (JAES) goes even further in nuancing their recommendations, with the note that the recommendations are based on low level evidence. For intermediate and high-risk PTC, they recommend even prophylactic lateral neck dissection in some cases, based on prognostic factors, such as age older than 55 years, male sex, significant extra-thyroidal extension, and tumor size measuring 3 cm or larger, but also taking into account the patient’s background and wishes [75]. These guidelines introduce the concept of risk factors for lateral neck involvement but also shared decision-making.

Finally, the French Speaking Association of Endocrine Surgery (AFCE) along with the French Associations of Endocrinology and of Nuclear Medicine, recommend selective neck dissection of levels III and IV if those levels are clinically involved and in the absence of clinical involvement of other neck levels. A prophylactic neck node dissection of sublevel IIB may be discussed, but only when there are proven metastatic nodes in sublevel IIA, due to the risk of spinal accessory nerve damage [76]. These recommendations state that prophylactic sublevel VB neck node dissection can be discussed when simultaneous positive nodes are proven in levels II, III or IV, thus taking into account risk factors when determining the extent of lateral neck dissection.

These discrepancies in various guidelines are due to the low-level evidence available and to the heterogeneity of the clinical presentation of cN1b PTC and varying philosophies regarding microscopically positive nodal disease. The analysis of our results highlights an uneven distribution of lymph node metastases in differentiated thyroid cancer, with significant variations across neck levels. Most studies included patients both with clinical lymph node metastases in each neck level but also those in whom clinically negative neck levels were dissected as well. Selected publications exhibited a certain degree of heterogeneity as some studies included cases of re-operation or pediatric patients, potentially affecting the results. Most studies did not include details as to the exact pathology of the cancers and may have included aggressive pathologic subtypes and follicular carcinoma along with classic papillary carcinoma, adding to the heterogeneity of the extent of neck involvement. The newest ATA guidelines thus distinguish papillary carcinoma and its variants from follicular carcinoma and its variants, due to distinct differences in the natural history of these lesions, including the incidence of lymph node metastases [2].

Discrepancies in classifying the exact location of lateral and central lymph nodes, particularly in borderline areas between contiguous neck levels, such as the retrocarotid area between levels IV and VI, may also have added to the heterogeneity of reported neck involvement.

Preoperative lymph node mapping has been shown to be susceptible to subjective variability in interpretation by radiologists [77]. However, certainly a case can be made given the heterogeneity of the literature for a personal nodal mapping prior surgery encompassing both ultrasound and CT scanning. This implies a patient-centered patient specific nodal map which can direct the surgery for an individual patient. This avoids reliance on systematic neck dissection and tailors surgery for clinically apparent and radiographically identifiable disease [77,78,79,80]. Further improvements in imaging technology will undoubtedly enable clinicians in the future to further tailor lymph node mapping and personalized surgery.

The boundaries between the central and lateral compartments (levels VI and IV) and the boundaries between lateral neck levels themselves are subject to interpretation. Neck nodes in between neck levels may be classified differently by different surgeons, as well. Furthermore, some studies suggest that, despite extensive neck dissection, patients with recurrence will recur in sites that are in between or outside of the defined anatomical boundaries of neck dissections [80].

There is a lack of strong evidence to show improved outcomes in terms of neck recurrence with extensive neck dissection versus a tailored approach, that is, there is no clear evidence that extending dissection to uninvolved adjacent lymph node regions improves prognosis or oncologic outcomes. Thus, within the framework of shared decision-making with the patient, and in accordance with recommendations that allow for some flexibility, the extent of lymph node dissection may be tailored based on individual factors such as age, comorbidities, and the burden of nodal disease.

An important aspect when considering the extent of therapeutic lateral neck dissection is the potential for morbidity, particularly spinal accessory nerve dysfunction. Sublevel IIB dissection has consistently been reported as the most relevant contributor to shoulder dysfunction. A “lower” VB dissection or “super-superselective” VB dissection that many surgeons prefer for papillary thyroid carcinoma may be an acceptable compromise between elective VB dissection and preservation of spinal accessory nerve function [81]. Unfortunately, few of the included studies in our meta-analysis provided systematic data on these complications, limiting the ability to draw conclusions. Future studies should report functional outcomes alongside oncological results to better inform surgical decision-making.

Our analysis of risk factors for lymph node metastases according to neck level (Table 2) highlights the heterogeneity of determinants across the lateral neck. Larger tumor size, macroscopic extranodal extension, and multifocality were consistently associated with increased risk, particularly for levels III and IV. Tumor location also emerged as a factor, with upper pole tumors more frequently metastasizing to sublevels IIA and IIB, and lower pole tumors showing a predilection for level V involvement. In addition, a higher burden of central lymph node metastases (≥3 nodes) correlated with more extensive lateral spread, especially in levels III–IV. Younger age (<55 years) and male sex were associated with higher risk in certain series, while specific histological variants (tall cell, hobnail) and molecular alterations, including BRAFV600E and TERT promoter mutations, have been linked to aggressive nodal disease, particularly in level V and sublevel VB. These findings underscore the importance of integrating both anatomical and molecular predictors into preoperative risk stratification to tailor the extent of therapeutic lateral neck dissection. In the future, artificial intelligence may aid in individual risk-factor analysis and decision-making. Further systematic reviews and meta-analyses may also aid in better understanding these risk factors and employ them to determine the optimal extent of neck dissection for a given patient.

One of the key limitations of our study lies in the methodology of literature review. The review was conducted manually without automated screening tools, potentially introducing selection bias. Another important limitation is that none of the included studies differentiated between more aggressive histological subtypes, which could have influenced the rate of lymph node metastases. What also remains unclear was the extent of radiologically involved levels in the studies and therefore whether teams who reported their data were dissecting levels expected to be involved or those which were considered disease free on pre-treatment imaging. The key question in the era of high definition imaging is whether levels of the lateral neck can be safely left undissected when contemporary scans suggest they are disease free. In addition, the size and therefore clinical importance of involved lymph nodes also remain unclear in the majority of studies limiting conclusions that can be drawn. It is known that the occult nodes that are present in many undissected necks in the setting of papillary thyroid carcinoma never progress to clinical disease. This underscores the problem of prophylactically extending lateral neck dissection to clinically uninvolved neck levels. Finally, this study did not analyze patient outcomes based on the extent of neck dissection, particularly comparing more extensive dissections that included sublevels IIB and VB, with more conservative surgical approaches.

## 6. Conclusions

Our meta-analysis of 57 studies suggests that the prevalence of lateral neck metastases in thyroid cancer follows a distinct pattern. Levels III (68%) and IV (66%) are most frequently involved, followed by level IIA (46%). In contrast, level I (6%) and sublevel VA (4%) are rarely involved. Sublevel IIB (14%) and level V (22%) overall show intermediate risk, though subanalysis reveals sublevel VB (19%) does carry a higher risk than VA.

Risk stratification, combined with key risk factors such as aggressive histology, molecular profile, and primary tumor characteristics, may help guide the extent of lateral neck dissection. A selective approach targeting levels IIA, III, IV +/− VB while often sparing level I, IIB, and VA can optimize oncologic control while minimizing morbidity. Ultimately, multidisciplinary, personalized decision-making is essential, and future prospective studies should validate this level-based risk stratification.

## Figures and Tables

**Table 2:** Risk factors for metastasis to specific nodal stations.

Neck Level	Risk Factors	References
**Level I**	- Macroscopic extranodal invasion	[12]
	- Multilevel neck disease in other levels	[12,13,14]
**Level II**		
**Sublevel IIA**	- Patients with extensive lymph node involvement and tumor location in the upper pole of the thyroid	[38,64]
	- More than 3 central lymph node metastases (CLNM)	[20,65]
**Sublevel IIB**	- A tumor larger than 20 mm	[23]
	- Large tumor size - Simultaneous lateral lymph node metastases in different levels	[20,47]
	- Tumor located in the upper pole of the thyroid	[38,64,66]
	- Metastases in levels III and IV	[67]
	- Extrathyroidal extension	[38]
	- Preoperative distant metastases	[38,68,69]
	- Larger tumor size	[40,70]
**Level III and IV**	- Extensive nodal involvement, particularly in level III	[38,68]
	- Tumor size ≥2.5 cm, number CLNM ≥ 3, level II metastases and BRAFV600E	[39]
	- Image-based, isolated lateral level IV involvement and macroscopic extranodal extension were independently associated with level II metastasis or either level II or V metastasis (*p* < 0.01). Macroscopic extranodal extension was also independently associated with level V metastasis (*p* = 0.001)	[31]
**Level V**	- Macroscopic extranodal disease extension in other levels	[30]
	- A higher number of central lymph node metastases significantly increases the risk of lateral lymph node metastases, including levels III and IV	[39,63]
	- Younger age (<55 years)	[29,63]
**Sublevel VA**	- Increased tumor size is consistently linked to a higher risk of lymph node metastases, and BRAFV600E mutation	[39]
	- Tumors located in the lower pole of the thyroid gland are particularly associated with level V metastases	[38,39,68]
	- Multiple metastatic lymph nodes at levels III and IV	[13,39]
	- Younger age	[29,71]
**Sublevel VB**	- Lymphovascular involvement	[31]
	- Male sex	[31]

## Data Availability

The data presented in this study are available on request from the corresponding author.

## Supplementary Materials

The following supporting information can be downloaded at https://www.mdpi.com/article/10.3390/diagnostics15202613/s1, The PRISMA 2020 checklist.
